# Unidirectional Ligament Orientation Enables Enhanced Out-of-Plane Mechanical Properties in Anisotropic Nanoporous Gold

**DOI:** 10.3390/nano15211675

**Published:** 2025-11-04

**Authors:** Yuhang Zhang, Xiuming Liu, Yiqun Hu, Suhang Ding, Feixiang Tang

**Affiliations:** 1 Hubei Digital Manufacturing Key Laboratory, School of Mechanical and Electronic Engineering, Wuhan University of Technology, Wuhan 430070, China; 2 Institute of Technological Sciences, Wuhan University, Wuhan 430072, China; liu_xiu_m@whu.edu.cn; 3 School of Integrated Circuits, Anhui University, Hefei 230601, China; 4 College of Mechanical Engineering, Donghua University, Shanghai 201620, China; dsuhang@dhu.edu.cn; 5 School of Microelectronics, Hefei University of Technology, Hefei 230009, China; thomas0209@hfut.edu.cn

**Keywords:** nanamechanics, nanoporous materials, mechanical properties, anisotropic behaviors, deformation mechanisms

## Abstract

Nanoporous gold (NPG), characterized by a bicontinuous network of nanoscale solid ligaments and pore channels, exhibits exceptional physical and chemical properties. However, the limited strength and stiffness of traditional isotropic NPG (INPG) have constrained its engineering applications. To effectively enhance the mechanical properties of NPG, this work proposes an innovative anisotropic NPG (ANPG) architecture featuring unidirectional ligament orientation. By controlling spinodal decomposition parameters, ANPG models with preferentially aligned ligaments and INPG with random ligament orientation are constructed, spanning relative densities from 0.30 to 0.50. The ligament length and diameter of ANPG along the out-of-plane direction are twice those along other directions. Molecular dynamics simulations of tensile tests show that ANPG exhibits superior out-of-plane Young’s modulus and yield strength but reduced fracture strain compared to INPG. Crucially, ANPG maintains toughness comparable to INPG at relative densities below 0.4, offering an optimal strength-toughness balance for practical applications. Scaling law analysis demonstrates INPG follows classical bending-dominated Gibson-Ashby behavior, while ANPG exhibits a hybrid deformation mechanism with significant ligament stretching contribution. Atomic-scale analysis reveals that both structures develop dislocation-mediated plasticity initially, but ANPG transitions to localized ligament necking and fractures more rapidly, explaining its reduced ductility. Strain localization quantification, measured by atomic shear strain standard deviation, confirms the intensifier deformation concentration in ANPG at large plastic strain. These findings suggest anisotropic design as a powerful strategy for developing high-performance NPG for actuators, sensors, and catalytic systems where simultaneous mechanical robustness and functional performance are required.

## 1. Introduction

Nanoporous metals (NPMs) have emerged as a unique class of functional materials that combine high specific surface area with remarkable physical, chemical, and mechanical properties, making them particularly suitable for advanced applications such as energy absorption, sensing, catalysis, microelectronics, and actuation [1,2,3,4]. However, the widespread practical implementation of conventional isotropic NPMs has been limited by their inherent mechanical deficiencies, particularly inadequate strength and stiffness that often fail to meet the demanding requirements of engineering applications [5,6,7]. This mechanical limitation becomes especially critical in precision devices where mechanical integrity must be maintained under operational stresses while preserving functional performance.

A comprehensive understanding of the mechanical properties and microscopic deformation mechanisms of NPMs serves as a fundamental prerequisite and key factor for designing their multiscale structures and enhancing mechanical performance. Researchers have conducted extensive investigations into the mechanical behaviors of NPMs through both experimental and modeling approaches. Experimental investigations by Song et al. [8] demonstrated that grain refinement through cyclic hot-rolling could significantly strengthen crack propagation resistance in nanoporous gold (NPG), with fine-grained structures exhibiting stress intensity factors three times higher than conventional coarse-grained counterparts. Studies by Loaiza et al. [9] systematically quantified the dominant influence of relative density on mechanical strength in nanoporous metallic foams with multiscale ligament structures. Another research group led by Zhang et al. [10] successfully decoupled the effect of surface triple junctions of NPG, revealing their crucial role in strengthening sub-100 nm scale ligaments through controlled dislocation emission. Furthermore, the implementation of multiscale structural design enables a significant reduction in the density of NPMs while preserving their high strength, primarily because this hierarchical architecture effectively prevents the loss of connectivity of solid ligaments at low relative densities [11,12,13]. Under compressive loading, these hierarchical NPMs typically exhibit a sequential deformation mechanism: larger ligaments undergo densification first, followed by the progressive collapse of smaller-scale ligaments [12].

Parallel advances in computational modeling have provided atomic-scale insights into deformation mechanisms of NPMs. For example, Liu and coworkers [14] investigated the microstructure evolution in nanoporous Au-Pt alloys, identifying how surface segregation and spinodal decomposition collectively influence the structural stability. Zandersons et al. [15] established comprehensive scaling relationships between Young’s modulus and solid volume fraction, emphasizing the concept and impact of “load-bearing solid fraction” in mechanical performance prediction. Molecular dynamics (MD) simulations of Yildiz et al. [16] revealed significant strain rate effects on nanocrystalline NPG, demonstrating enhanced strength and toughness at higher loading rates accompanied by complex deformation mechanisms including grain rotation and boundary sliding. Most recently, Li et al. [17] proposed an innovative core-shell structure NPG@Pt, where interfacial stress-induced dislocation interactions effectively suppress strain localization and substantially improve material plasticity. They also studied the mechanical behaviors of NPG under multiaxial tension and found that the failure of NPG under multiaxial tension is governed by the progressive yielding, necking, and rupture of ligaments along multiple loading directions [18]. Their research [18] further revealed that dislocation activity under multiaxial stress states is substantially more intense compared to uniaxial conditions, leading to enhanced plastic deformation susceptibility and ultimately resulting in reduced strength and failure strain. Beyond revealing the quasi-static deformation processes of NPMs, MD simulations have also been extensively employed to investigate their dynamic deformation mechanisms. Saffarini et al. [19], through MD simulations, revealed that NPG significantly alters shock wave propagation behaviors through pore collapse and ligament fragmentation mechanisms under high-velocity impact. Unlike solid gold targets, the heterogeneous stress field generated by NPG causes complete failure of downstream protected specimens, demonstrating the dual nature of energy dissipation and stress dispersion in porous materials under impact loading [19]. In addition, based on large-scale MD simulations of NPG under compressive loading [20], a dislocation-based multiscale constitutive framework is established that connects atomic-scale deformation mechanisms with continuum-level plasticity models. This approach generates physics-grounded datasets for AI-aided design of architected NPMs with programmable mechanical properties across scales [20].

NPG, with its feasible fabrication process and superior properties, serves as an exemplary model system for investigating the fundamental properties and behaviors of NPMs. The practical applications of NPG have been substantially constrained by its inherent limitations in mechanical strength and toughness. Recent advances in several types of porous materials have demonstrated that unidirectional architectural design of solid ligament networks can remarkably enhance the mechanical performance of porous materials [21,22,23,24]. Inspired by these developments, the present study presents an anisotropic nanoporous gold (ANPG) featuring unidirectional aligned ligaments, where the out-of-plane ligament length and diameter are twice those in the in-plane directions. The mechanical properties and deformation mechanisms of this unique structure are systematically investigated through MD simulations. It is found that the anisotropic structural design can simultaneously enhance stiffness and strength while maintaining adequate toughness at low relative densities, opening new possibilities for developing advanced nanoporous materials for next-generation sensors, actuators, and other precision engineering applications.

## 2. Models and Method

### 2.1. Sample Generation

In this work, the classical Cahn-Hilliard equation [25] is solved to simulate the spinodal evolution process of two phases and construct the INPG and ANPG samples with various relative densities (porosities):

(1) $$\frac{\partial u}{\partial t}=\nabla^{2}\left[\frac{df(u)}{du}-\theta^{2}\nabla^{2}u\right]$$

where ∇ is the gradient operator, *u* is the concentration difference of the two phases (void and solid Au ligament in this work) and *u* is a function of spatial location (*x*, *y*, *z*) and system running time *t*, *f*(*u*) is the free energy function, and θ is the transition region width between the solid regime and void regime. Herein, $f(u)={\left(u^{2}-1\right)}^{2}/4$ and θ=0.01 are determined according to previous work [26]. We use a 200 (*x*) × 200 (*y*) × 100 (*z*) cubic grid to construct the initial solid geometric model of NPG samples. It should be noted that each grid is denoted by a point coordinate (*ib*, *jb*, *kb*), where *b* is the mesh size and (*i*, *j*, *k*) is the serial number of the grid in three-dimensional space (i.e., from (0, 0, 0) to (199, 199, 99)). The mesh size is set as 0.1 [27]. The finite difference method (FDM) is used to solve Equation (1), as described in the reference [26]:

(2) $$\frac{u_{ijk}^{m+1}-u_{ijk}^{m}}{\tau }=\nabla^{2}\left[{\left(u_{ijk}^{m}\right)}^{3}-u_{ijk}^{m}-\theta^{2}\nabla^{2}u_{ijk}^{m}\right]$$

(3) $$\nabla^{2}u_{ijk}^{m}=\frac{u_{(i+1)jk}^{m}+u_{(i-1)jk}^{m}+u_{i(j+1)k}^{m}+u_{i(j-1)k}^{m}+u_{ij(k+1)}^{m}+u_{ij(k-1)}^{m}-6u_{ijk}^{m}}{b^{2}}$$

where $u_{ijk}^{m}$ is the concentration difference of the two phases at the node point (*ib*, *jb*, *kb*) when the system evolution time is *mτ*, *τ* is the integration time step and is set as 0.001 in this paper, and *m* is an integer representing the number of discrete time step that have evolved. The initial concentration difference in each grid is randomly distributed within the range $[-1,\;1]\times 10^{4}$, with periodic boundary conditions (PBCs) adopted in all three Cartesian directions [27]. With increasing system operation time, the ligaments coarsen continuously, which is consistent with experimental investigation [28]. When the system runs 40,000 steps, namely *m* = *m*_t_ = 40,000, the instant state of the 3D concentration distribution is recorded and used to fabricate the geometric sample of NPG. For each node point (*ib*, *jb*, *kb*), if $u_{ijk}^{m_{t}}>u_{c}$ the representing grid is solid; if $u_{ijk}^{m_{t}}\leq u_{c}$, the grid is void. Here, *u*_c_ is the critical concentration difference that determines the solid fraction of the sample. By altering *u*_c_, the relative density of the porous specimen can be adjusted. Subsequently, the dimensions are scaled to produce a solid geometric model with targeted sizes.

**Figure 1:**
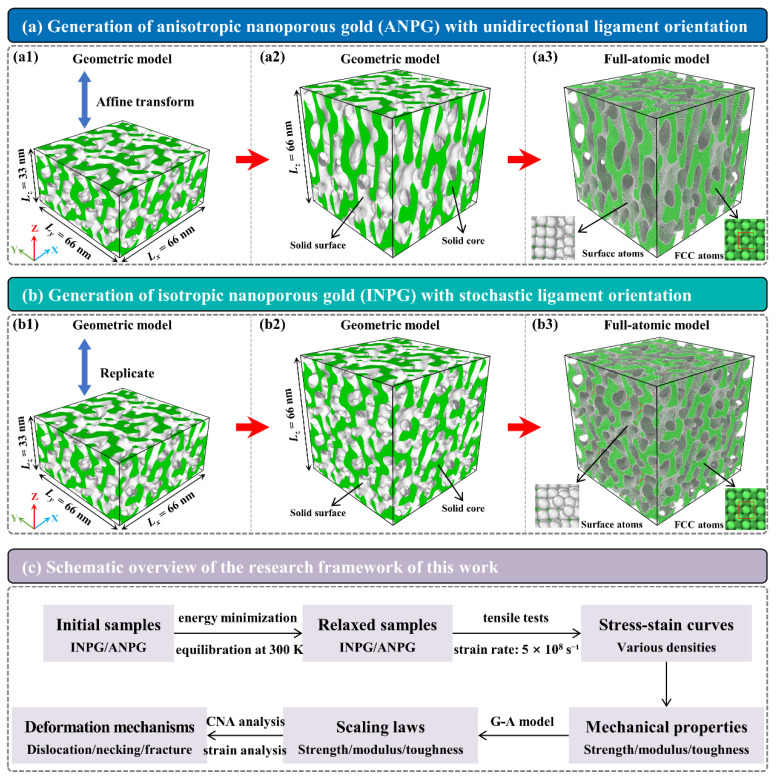
Sample generation of (**a1**–**a3**) anisotropic NPG (ANPG) sample with unidirectional ligament orientation and (**b1**–**b3**) isotropic NPG (INPG) sample with stochastic ligament orientation. The sample has a relative density of *ρ* = 0.40. (**c**) A schematic overview of the research framework of this work.

Figure 1 gives the fabrication processes of the full-atomic model of the anisotropic/isotropic NPG with a relative density (*ρ*) of 0.40. As Figure 1(a1,b1) show, the initial NPG sample is generated by solving the Cahn-Hilliard equation, with the dimensions of 66 nm (*L_x_*) × 66 nm (*L_y_*) × 33 nm (*L_z_*). The dimensions in the *x*- and *y*-directions are set to be twice that of the *z*-direction, such that the subsequent preparation of two types (unidirectional and stochastic ligament orientations) of nanoporous samples is enabled. As Figure 1(a1,a2) illustrate, the NPG sample with initially anisotropic dimensions undergoes an affine transformation along the *z*-direction, in which the *z*-coordinates are scaled by a factor of two, resulting in a final structure with identical three-dimensional dimensions of 66 nm (*L_x_*) × 66 nm (*L_y_*) × 66 nm (*L_z_*) (Figure 1(a2)). The transformed material exhibits anisotropic characteristics, with ligaments preferentially aligned along the *z*-direction (out-of-plane direction). Specifically, the length and thickness of the ligaments in the *z*-direction are twice those in other directions. Differently, when the initial sample is duplicated once along the *z*-direction, as denoted in Figure 1(b1,b2), the resulting NPG structure becomes isotropic. In this case, the ligament orientations are random, and their length and width are uniformly distributed in all three directions, indicating isotropic behavior. Note that the structures generated in Figure 1(a2,b2) are solid geometric models. The white regions represent the interface between the solid ligaments and the pores, while the green areas represent the interior of the solid ligaments, with 3D PBCs applied. The geometric model serves as a template to construct the final full-atom model of NPG. The parent material is defined as face-centered cubic (FCC) single-crystal gold with crystal orientations along [001], [010], and [100]. Topological operations are performed on the geometric template to generate the atomic structure by the software ATOMSK Beta 0.13.1 [29] (atoms located outside the surface are deleted to create the surface). In the resulting model, as illustrated in Figure 1(a3,b3), green atoms correspond to the regular FCC lattice, and white atoms represent disordered surface atoms.

**Figure 2:**
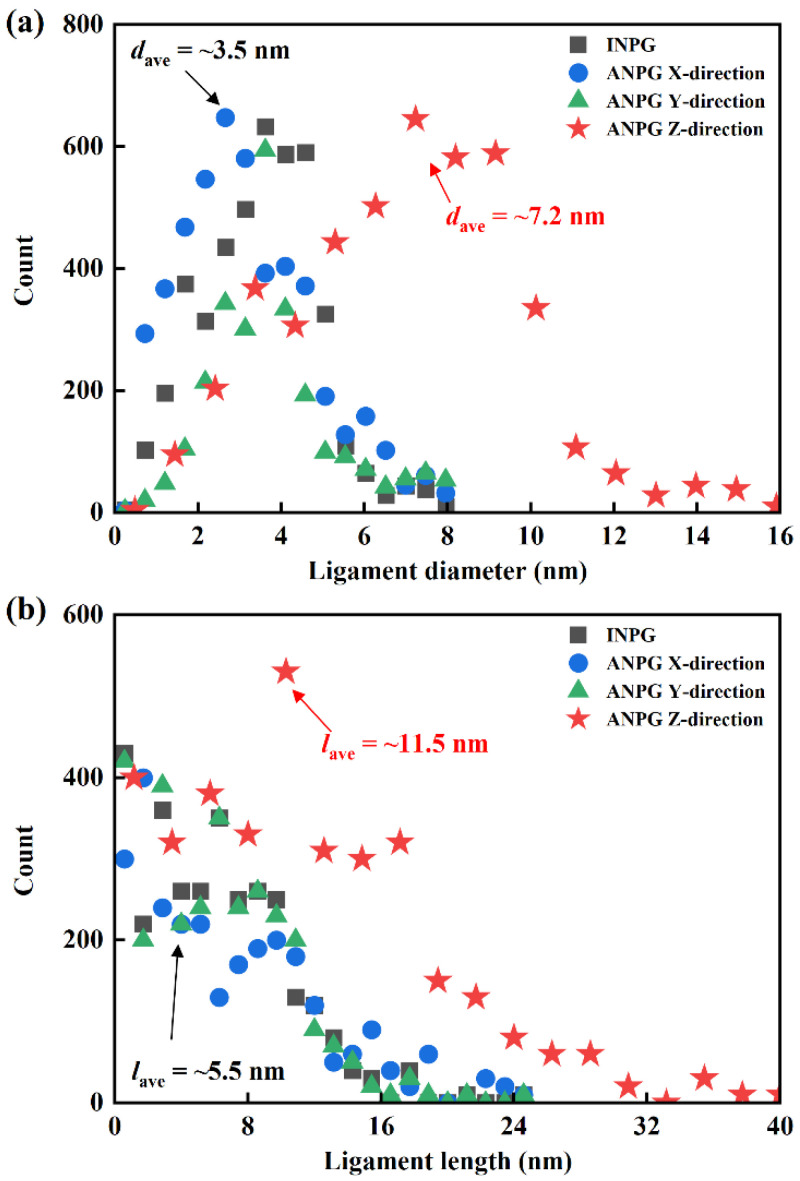
Distribution of (**a**) ligament diameter and (**b**) ligament length of the generated INPG and ANPG samples. Note that due to the unidirectional ligament orientation of the ANPG, its distribution of ligament size is statistically analyzed individually along three different directions.

To characterize the ligament size distribution of the generated full-atomistic models, we employ the AQUAMI 1.0 [30] software package to statistically analyze the distributions of ligament diameter and length of the NPG samples with a relative density of 0.40, as shown in Figure 2. For the isotropic NPG (INPG), the ligament size distribution is consistent along all three Cartesian directions due to its structural homogeneity. In contrast, for the anisotropic NPG (ANPG), which exhibits ligament alignment preferentially along the *z*-direction, the ligament size distributions are characterized separately for each Cartesian direction. As illustrated in Figure 2a, the ligament diameter distribution of the INPG sample exhibits a peak at a median value with tails on either side, resembling a Gaussian or log-normal distribution, which is consistent with findings reported in the literature [31,32,33]. The ligament diameter distribution of ANPG along the *x* and *y*-directions is similar in range and intensity to that of INPG. However, the distribution for the *z*-direction is noticeably shifted to the right, indicating a larger overall ligament diameter. Quantitative analysis reveals that the average ligament diameter (*d*_ave_) is approximately 3.5 nm for INPG, while it is about 7.2 nm along the *z*-direction for ANPG. A similar trend is observed for ligament length. As indicated in Figure 2b, the distribution of ligament length in the *z*-direction of the ANPG is noticeably shifted to the right compared with that in the INPG, indicating a larger overall ligament length. Specifically, the average ligament length (*l*_ave_) of ANPG along the *z*-direction is approximately twice that of INPG (11.5 nm and 5.5 nm). This result is a direct consequence of our modeling approach, where the ANPG structure is generated via an affine transformation that specifically doubled the ligament dimensions along the *z*-direction.

### 2.2. MD Simulations of Mechanical Tests

To investigate the influence of relative density on the mechanical properties and to determine whether the mechanical scaling laws of the ANPG align with those of traditional nanoporous materials, we construct full-atomic models with five different relative densities: *ρ* = 0.30, 0.35, 0.40, 0.45, and 0.50. For each relative density, both ANPG and INPG models are modeled, with all samples sharing identical global three-dimensional dimensions. The average ligament sizes across the different models are maintained nearly constant (variation < 10%) by adjusting the system running time step and the cutoff value in the Cahn-Hilliard spinodal decomposition equation. As the relative density increases from 0.30 to 0.50, the total number of atoms in the simulation system correspondingly rises from approximately 4.85 million to 8.19 million.

The overall research flow of this work is shown in Figure 1c. Specifically, upon completion of the atomistic model construction, energy minimization is performed using the widely used conjugate gradient (CG) algorithm to ensure a stable initial configuration. The minimized structure subsequently undergoes a thermal equilibration procedure in the isothermal-isobaric (NPT) ensemble, maintained at a room temperature of 300 K and 0 GPa in all three directions for 500 ps, allowing the system to reach thermodynamic equilibrium under ambient conditions. Following adequate equilibration, MD simulations of mechanical tests are conducted to evaluate the mechanical responses of the ANPG and INPG samples with various relative densities. Uniaxial tensile deformation is applied along the *z*-direction with a constant engineering strain rate of 5 × 10^8^ s^−1^, while the lateral dimensions (*x* and *y*-directions) are stress-controlled to remain at 0 GPa, effectively simulating a uniaxial stressed state. This strain rate of 5 × 10^8^ s^−1^, while higher than experimental quasi-static rates, is common practice in MD simulations due to the limitations of timescale [34,35,36]. It is well-established that such a strain rate can accurately capture the fundamental deformation mechanisms and qualitative mechanical trends in nanostructured materials [37,38,39]. Throughout all simulation stages, full three-dimensional PBCs are imposed to eliminate spurious surface effects. After obtaining the stress-strain curves, the mechanical properties are calculated, and mechanical scaling laws are developed. Subsequently, the deep analysis of microstructure evolution is performed by tracking the variation in atomic types and local atomic strain.

## 3. Results and Discussion

### 3.1. Stress-Strain Responses

**Figure 3:**
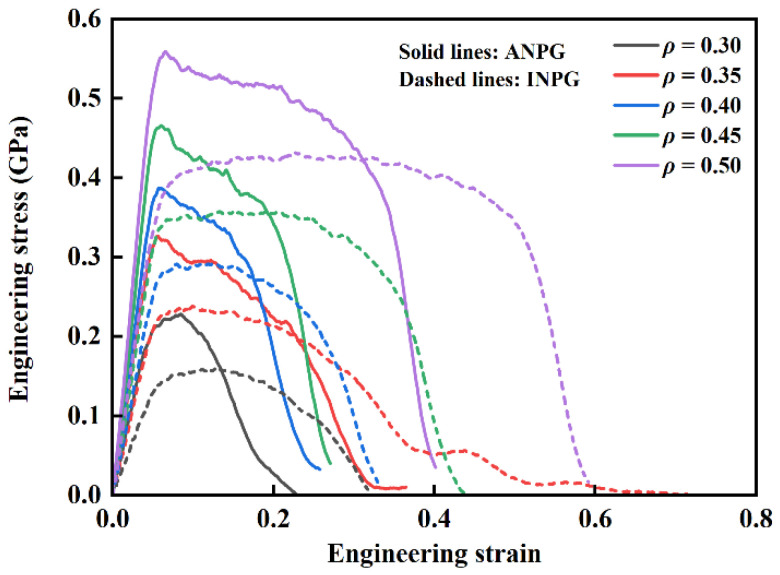
Uniaxial tensile engineering stress-strain curves of ANPG and INPG with varying relative densities, obtained at 300 K and an engineering strain rate of 5 × 10^8^ s^−1^. Solid and dashed lines represent the mechanical responses of ANPG and INPG samples, respectively.

In this subsection, we first discuss the stress-strain responses of INPG and ANPG atomistic samples with varying relative densities under tension. Figure 3 shows the typical uniaxial tensile engineering stress-strain diagram of ANPG and INPG samples with varying relative densities, obtained at 300 K and an engineering strain rate of 5 × 10^8^ s^−1^. Note that the solid and dashed lines represent the mechanical responses of ANPG and INPG, respectively. Both the ANPG and INPG exhibit tensile deformation characteristic that aligns well with the established mechanical response patterns of traditional nanoporous metallic materials obtained by MD simulations [16,18,38]. The entire stress-strain responses can be divided into three consecutive deformation stages: the initial linear elastic deformation stage, the subsequent yielding and plastic flow plateau stage, and the final rapid crack propagation and failure stage. In the first linear elastic regime, the applied stress demonstrates a rapid, linearly proportional increase with the accumulating engineering tensile strain, following the classic Hooke’s law. Upon reaching the critical yield point, a noticeable transition occurs where further strain accumulation no longer produces a corresponding stress increase; instead, the stress level becomes suppressed, forming a distinct flow plateau where the stress remains nearly constant or undergoes a gradual, moderate decrease while strain continues to accumulate significantly. The final stage commences when localized ligament fracture initiates microcrack formation, creating stress concentration sites that drive rapid crack propagation through the whole structure, ultimately leading to an abrupt, dramatic stress drop that culminates in complete structural failure.

When comparing INPG and ANPG systems quantitatively, distinct differences emerge in their mechanical responses despite sharing the same general stress-strain features. Throughout the initial linear elastic deformation phase and the early yielding transition, the stress-strain curves of ANPG appear above those of INPG with identical relative densities, unambiguously indicating superior elastic modulus and yield strength of ANPG. In specific, at relative densities of 0.30, 0.35, 0.40, 0.45, and 0.50, the Young’s moduli of ANPG reach 4.62, 6.66, 7.59, 9.08, and 10.71 GPa, respectively, corresponding to 84.8%, 89.2%, 73.7%, 53.6%, and 55.2% increases over INPG (2.50, 3.52, 4.37, 5.91, 6.90 GPa); the yield strength of INPG is 0.16, 0.23, 0.29, 0.35, and 0.43 GPa, while that of ANPG reaches 0.23, 0.32, 0.38, 0.46, and 0.56 GPa, representing significant enhancements of 43.8%, 39.1%, 31.0%, 31.4%, and 30.2% over INPG at corresponding relative densities. The enhanced mechanical properties of the ANPG along the out-of-plane direction can be attributed to the unidirectional solid frame acting as a support against external axial stress in this direction [23]. In addition, the mechanical properties of ANPG demonstrate significantly greater enhancement compared to INPG at lower relative densities, with the improvement in stiffness being markedly more substantial than that in strength. This characteristic performance advantage positions ANPG as particularly suitable for applications in sensors and actuators. However, this strength advantage comes with a noticeable trade-off in deformation ability during the plastic flow regime. ANPG specimens demonstrate a considerably shortened plastic flow duration, exhibit more pronounced stress decline during this stage, and transition more rapidly into the final failure phase. Specifically, for a representative case with 0.5 relative density (purple dashed line in Figure 3), INPG maintains an exceptionally stable flow stress with minimal variation until approximately 0.5 engineering strain, after which an abrupt stress drop leads to final fracture at around 0.6 strain. In contrast, ANPG at the same relative density of 0.5 already shows significant stress deterioration by 0.3 strain (purple solid line in Figure 3), with complete fracture occurring at approximately 0.4 strain. This pattern remains consistent across all investigated relative densities. The results in Figure 3 suggest that while ANPG possesses enhanced elastic modulus and yield strength in the out-of-plane direction, it achieves these properties at the expense of reduced ductility, manifested through a narrower plastic flow strain range and significantly lower fracture strain compared to its isotropic counterpart.

### 3.2. Scaling Laws of Mechanical Properties

**Figure 4:**
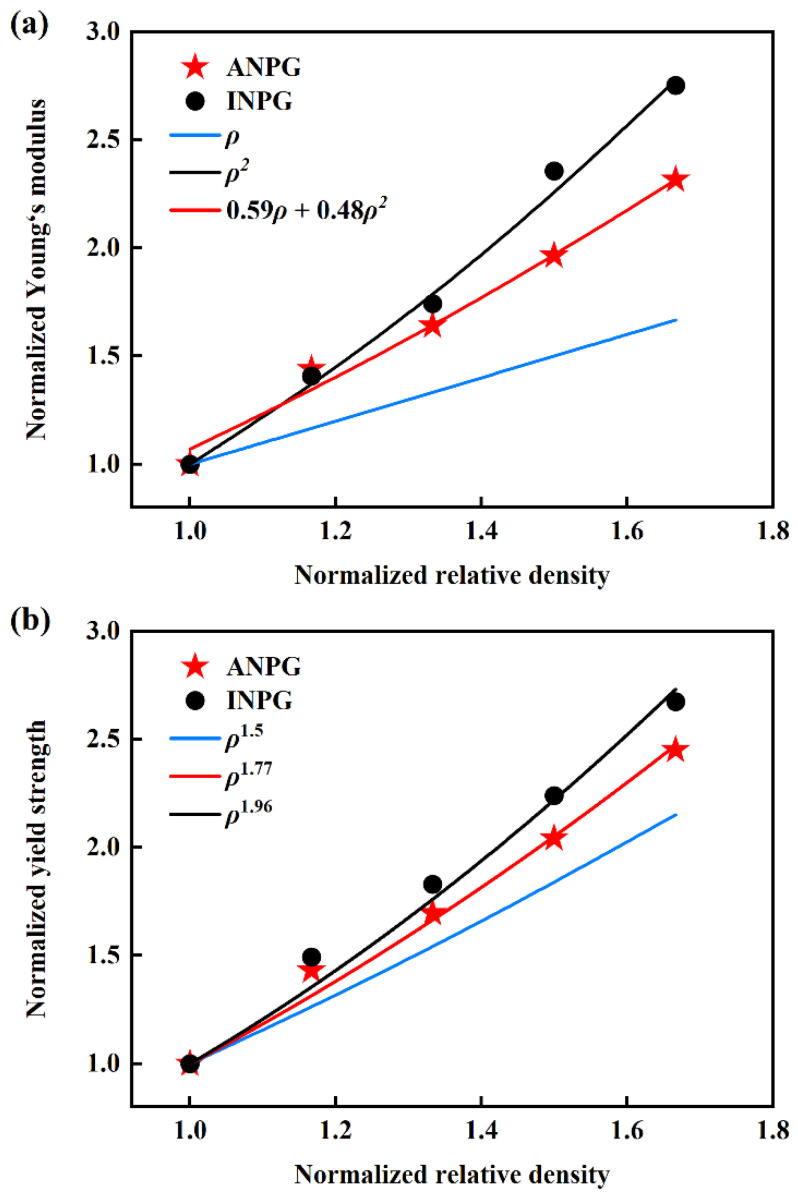
Normalized (**a**) Young’s modulus and (**b**) yield strength of the investigated INPG and ANPG as functions of relative density. All mechanical properties and relative densities have been normalized with respect to the corresponding values of the sample with 0.30 relative density, serving as the reference, to facilitate clearer investigation of the mechanical scaling relationships. The data points represent the mechanical properties obtained from MD simulations, while the lines correspond to theoretical predictions or fitting results.

A comprehension of the scaling relationships of mechanical properties in nanoporous materials not only provides crucial guidance for their structural design but also offers profound insights into the microscopic deformation mechanisms behind macroscopic mechanical behaviors. The classical Gibson-Ashby (G-A) theory [40,41,42] establishes fundamental scaling laws between modulus and relative density: when Young’s modulus *E* shows linear proportionality with relative density *ρ*, it indicates that the elastic deformation is primarily governed by the axial stretching of ligaments, whereas when *E* demonstrates quadratic proportionality with *ρ*, it signifies that the elastic deformation is dominated by ligament bending. Figure 4 demonstrates the mechanical properties as a function of the relative density of the tested samples. It is particularly important to note that the mechanical properties and relative densities presented in Figure 4 have been normalized with respect to the sample with 0.30 relative density, serving as a consistent baseline to facilitate clearer investigation and more straightforward interpretation of the mechanical scaling relationships. As Figure 4a denotes, we examine the relationship between Young’s modulus and relative density for both ANPG and INPG, with the classical G-A theoretical framework provided as a reference for comparative analysis. For INPG specimens (represented by black points in Figure 4a), the Young’s modulus *versus* relative density relationship exhibits remarkable agreement with the typical G-A model for bending-dominated structures ($E\propto \rho^{2}$, black line in Figure 4a), suggesting that the elastic deformation mechanism of INPG is predominantly governed by ligament bending/rotation, which aligns with the deformation characteristics observed in most conventional NPMs [15,43,44,45]. A previous experimental study by Hodge et al. [41] demonstrated that the Young’s modulus of NPG is influenced not only by relative density but also by ligament size. They further showed that the G-A model remains applicable when ligament size effects are properly accounted for [41]. Badwe et al. [46] found the Young’s modulus of NPG obeys a power law with relative density, but with an exponent larger than that predicted by the G-A scaling law, which might be attributed to the relatively larger relative density range (0.30–0.57). Li et al. [47], through MD simulations, reported that the Young’s modulus of NPG obeys the classical G-A model $E\propto \rho^{2}$. Herein, we have used normalized modulus in our analysis, thereby effectively eliminating the influence of ligament size and allowing us to focus specifically on the role of relative density.

For the ANPG system, the scaling behavior of elastic modulus deviates significantly from the predictions of the classical G-A law, with the experimental data points (red stars in Figure 4a) positioned between the theoretical lines for linear and quadratic proportionality, indicating a complex deformation mechanism where both ligament bending and axial stretching contribute concurrently to the overall elastic response. To quantitatively describe this hybrid elastic deformation behavior, we incorporate the modified scaling model developed by Sun et al. [26], which simultaneously accounts for both bending and stretching contributions to elastic deformation, and our analysis confirms that the elastic modulus of ANPG follows the comprehensive formula $E\propto (A_{1}\rho^{2}+A_{2}\rho^{2}),\;A_{1}=0.59,\;A_{2}=0.48$. The determined parameters show $A_{1}>A_{2}$, clearly demonstrating that axial stretching plays a more dominant role than bending throughout the elastic deformation process. This fundamental shift in elastic deformation mechanism can be directly attributed to the structural characteristics of ANPG where, due to our affine transformation processing, ligaments are not completely randomly oriented but instead show preferential alignment along the longitudinal direction, with ligaments along this orientation having approximately twice the length compared to those in other directions, and this strategically engineered anisotropy not only alters the deformation pathway but also provides the underlying structural reason for the enhanced elastic modulus and yield strength observed in ANPG compared to its isotropic counterpart.

According to the G-A theory for metallic foams, the yield strength $\sigma_{s}$ is expected to scale with relative density raised to the power of 1.5 [24]. As illustrated in Figure 4b, we present normalized yield strength versus relative density data for both INPG and ANPG architectures, revealing notable deviations from the classical G-A scaling law across the investigated density spectrum. Specifically, the MD data demonstrate a distinct power-law relationship characterized by exponents significantly larger than the theoretical value of 1.5 for both ANPG and INPG. Jeon et al. [11] experimentally tested the yield strength of NPG, and they found that the variation in yield strength with relative density is significantly greater than the G-A prediction, which is consistent with our results. This observed enhancement in scaling exponents can be primarily attributed to the relatively broader range of relative densities investigated in our study, spanning from 0.30 to 0.50, which encompasses both the low-density and moderate-density regimes. At lower relative densities, particularly approaching the percolation threshold, the structural connectivity between ligaments experiences considerable reduction, creating localized stress concentration sites and diminishing load transfer efficiency throughout the ligament network [20,31,32], thereby rendering the yield strength more sensitive to density variations and consequently producing steeper scaling relationships manifesting as higher exponent values. The results in Figure 4b demonstrate that the yield strength scaling relationships for INPG and ANPG follow $\sigma_{s}\propto \rho^{1.96}$ $\sigma_{s}\propto \rho^{1.77}$, respectively. Notably, the smaller exponent observed in ANPG indicates that its yield strength exhibits relatively less degradation at lower relative densities compared to conventional NPG. This phenomenon originates from the inherent microstructural features in the ANPG. Herein, the affine transformation is used in generating ANPG, which elongates ligaments along the out-of-plane direction (Figure 2) and thus improves ligament connectivity effectively, particularly at low densities. While stochastic INPG structures experience a rapid loss of load-bearing paths as density decreases due to the decreased ligament connectivity [12,15], the solid ligament network in ANPG preserves more continuous stress transmission channels. Consequently, the yield strength of ANPG degrades more gracefully under density reduction, making it particularly advantageous for lightweight applications where structural integrity must be maintained across varying porosity levels.

**Figure 5:**
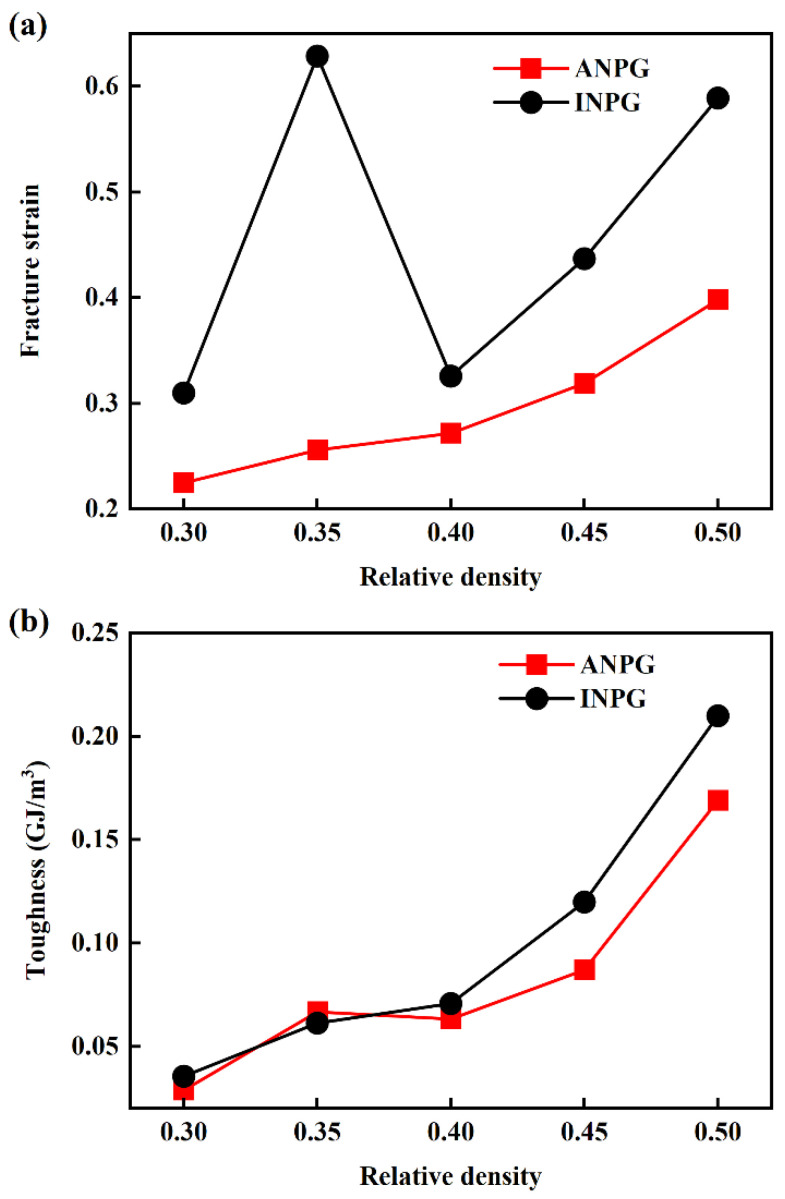
(**a**) Fracture strain and (**b**) toughness as a function of relative density for the tested ANPG and INPG.

Figure 5a illustrates the variation in fracture strain with relative density for the INPG and ANPG samples. As shown in Figure 5a, the fracture strain generally increases with rising relative density, with the exception of INPG at 0.35 relative density. This anomaly may be attributed to the high intrinsic ductility of individual nanoscale ligaments combined with the stochastic nature of failure in nanoporous structures composed of numerous interconnected nanoligaments, leading to scatter in macroscopic fracture strain data. Furthermore, at equivalent relative densities, INPG consistently exhibits higher fracture strain than ANPG. The area under the stress-strain curve represents the energy absorbed during tensile deformation [48,49,50], serving as a direct indicator of material toughness, i.e., the ability to resist fracture under tensile loading. Figure 5b presents the relationship between toughness and relative density, showing that toughness likewise increases with relative density. At relative densities up to 0.4, INPG and ANPG demonstrate comparable toughness, whereas above this threshold, INPG exhibits superior toughness compared to ANPG at equivalent densities, due to its higher fracture strain. Given that toughness is a critical metric for evaluating a material’s capacity to absorb energy before fracture. Our investigation into the toughness of both INPG and ANPG across a range of relative densities provides pivotal insights for selecting and designing NPG in energy-absorbing applications. Notably, the finding is that ANPG retains toughness comparable to INPG at relative densities below 0.4, while simultaneously offering superior strength and stiffness. This advantageous property stems from the robust, unidirectionally aligned ligament network of ANPG. Given that most experimentally fabricated NPG structures exhibit relative densities lower than 0.40, this finding provides a clear and practical guideline for material selection and design. Engineers can leverage the anisotropic architecture of ANPG to achieve excellent mechanical performance in applications such as sensors and actuators without incurring the typical penalty of reduced damage tolerance. This optimal strength-toughness balance positions ANPG as a compelling material candidate for next-generation lightweight and robust nanoscale devices.

### 3.3. Microstructural Deformation Mechanisms

**Figure 6:**
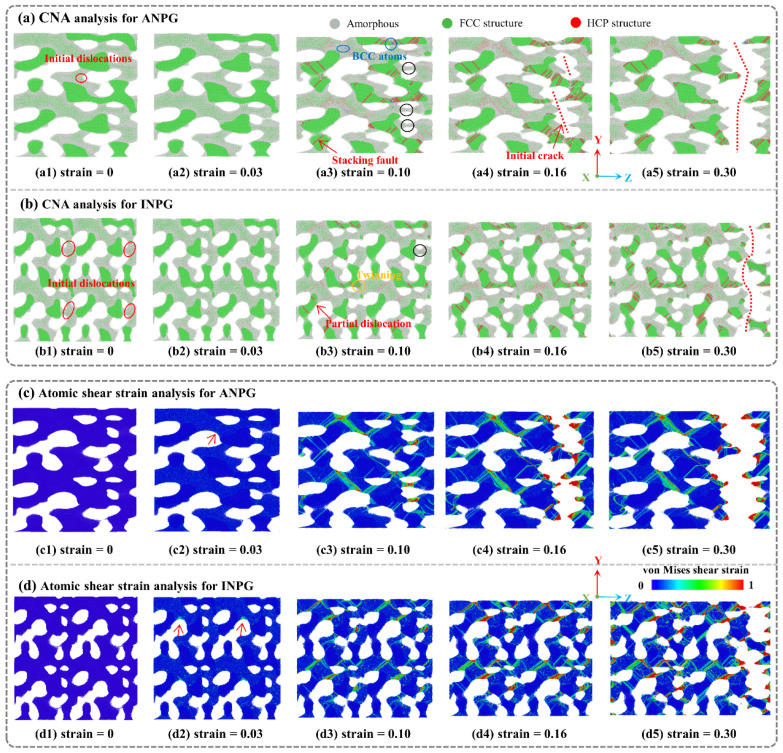
A sequence of snapshots showing the microstructural evolution in (**a**,**c**) ANPG and (**b**,**d**) INPG with a relative density of 0.40. Atoms are colored by their (**a**,**b**) lattice types and (**c**,**d**) von Mises shear strain values. Note that to facilitate clearer visualization of deformation details, only a representative 4 nm-thick slice along the *x*-direction is displayed.

Having clarified the mechanical properties and scaling relationships, we now investigate the microscopic deformation mechanisms of ANPG and INPG. MD simulations offer the distinct advantage of enabling in-depth analysis of material structural evolution from an atomic perspective. As shown in Figure 6a, we employ common neighbor analysis (CNA) [51] to study the atomic structure evolution during deformation in both structures. Atoms are color-coded according to their lattice types: green represents FCC-structured Au atoms, white corresponds to amorphous atoms (surface atoms), red indicates HCP-structured atoms, and blue denotes BCC-structured atoms. A single layer of HCP atoms signifies a twin boundary, a double layer represents a stacking fault, and larger HCP clusters indicate the formation of HCP phases [52]. Note that to facilitate clearer visualization of deformation details, only a representative 4 nm-thick slice along the x-direction is displayed. Figure 6a reveals that the ligament deformation processes and atomic structure transformations are generally quite similar in ANPG and INPG. At zero strain, before applied elongation deformation, nearly all atoms within the ligaments maintain perfect FCC structure, while surface atoms, due to reduced coordination, exhibit amorphous configurations. A minimal presence of HCP atoms resulting from initial thermal equilibration indicates pre-existing defects, as highlighted by the red ellipses at zero strain in Figure 6(a1,b1). As external uniaxial tensile strain is progressively applied, the structure undergoes continuous deformation. When the engineering tensile strain reaches 0.03, the structural response remains within the small elastic deformation regime, with no occurrence of new plastic deformation. As clearly demonstrated in Figure 6(a2,b2) at strain = 0.03, no new HCP atoms are observed at this stage, indicating that dislocation nucleation has not been activated, a characteristic signature of purely elastic deformation. Throughout this phase, the dominant deformation behavior primarily consists of individual Au nanowire bending deformation (for INPG) and combined bending-tension deformation modes (for ANPG), where ligaments elastically redistribute stresses without undergoing permanent structural changes. The absence of lattice transformation and defect generation confirms that the energy input is primarily stored as reversible elastic strain energy, maintaining the structural integrity of the nanoporous framework. As shown in Figure 6(a3,b3), at strain = 0.10, the two structures have undergone global yielding and entered the plastic flow stage. A significant number of HCP atoms newly emerge, indicating substantial plastic deformation. Specifically, partial dislocations are activated from the ligament surfaces and propagate continuously until they traverse the entire ligament, forming stacking faults characterized by two adjacent layers of HCP atoms, as marked by the red arrows in Figure 6(a3,b3) at strain = 0.10. Additionally, twinning phenomena and FCC-BCC phase transformations are observed, highlighted by red and yellow circles, respectively. However, the most critical deformation mechanisms remain the nucleation and motion of dislocations and the formation of stacking faults. For ANPG, intensive dislocation slip and interactions lead to pronounced ligament necking at the weakest regions, where the ligament diameter decreases sharply, as indicated by the black circles in Figure 6(a3). This localized necking induces structural softening and compromises the overall load-bearing capacity. Although ligament necking is also observed in INPG, its extent is significantly less pronounced than in ANPG (Figure 6(b3)). This difference in necking behavior underscores the distinct deformation responses between the anisotropic and isotropic nanostructures under plastic strain. At 0.16 strain, multiple ligaments in the ANPG structure have already fractured, forming initial microcracks as indicated by the red dashed lines in Figure 6(a4) at strain = 0.16. These microcracks serve as stress concentration points that promote rapid crack propagation, ultimately leading to complete structural failure. In contrast, under the same strain level of 0.16, the INPG structure shows no evidence of microcrack formation, allowing it to continue sustaining mechanical load, which corresponds to its extended plastic flow deformation stage observed in the stress-strain response. Finally, at the tensile strain of 0.30, cracks propagate throughout the entire observed slice, resulting in its final fracture. Additionally, during progressive deformation, ligament bending and reorientation might induce localized crystallographic misalignment. This misorientation effectively hinders dislocation transmission across the bent interfaces while promoting dislocation annihilation at the opposing free surfaces. The resulting impedance to plastic flow initiated progressive dislocation accumulation and saturation of stacking fault densities within the constrained ligament segments [6].

Furthermore, we analyze the distribution of equivalent atomic shear strain during the deformation process using the following formula [53]:

(4) $$\eta_{\mathrm{Mises}}=\sqrt{\eta_{yz}^{2}+\eta_{xz}^{2}+\eta_{xy}^{2}+\frac{{\left(\eta_{yy}-\eta_{zz}\right)}^{2}+{\left(\eta_{xx}-\eta_{zz}\right)}^{2}+{\left(\eta_{xx}-\eta_{yy}\right)}^{2}}{6}}$$

where $\eta_{\mathrm{Mises}}$ indicates the von Mises shear strain of a single atom and $\eta_{xy}$ is the strain tensor. The von Mises shear strain is an important tool to study local deformation [54]. As shown in Figure 6c,d, we observe in real time the evolution of atomic shear strain during tension deformation. In the absence of applied strain, no internal deformation is detected. When the strain reached 0.03, the two structures remain in the elastic deformation regime, with only minimal atomic strain localized at ligament surfaces or regions of stress concentration, as indicated by the red arrows in Figure 6(c2,d2). This localized strain distribution corresponds to the initial stress redistribution within the ligament network before global yielding, demonstrating how elastic deformation is accommodated through localized atomic displacements in these nanostructured materials. At the strain of 0.10, both the INPG and ANPG structures exhibit extensive plastic deformation, as clearly illustrated in Figure 6(c3,d3). In the ANPG, severe shear strain localization develops. Numerous localized shear deformation zones emerge, predominantly concentrated along active slip planes and within ligament necking regions. Subsequent rapid necking of these critically strained ligaments in ANPG intensifies the deformation concentration in adjacent regions, ultimately triggering failure reminiscent of crack propagation. Conversely, the INPG demonstrates fundamentally distinct strain distribution characteristics. A more homogeneous redistribution of shear strains occurs across a broader population of ligaments throughout the structural network at 0.10 strain. This deformation mechanism effectively suppresses localized failure initiation and enhances global ductility. No remarkable ligament necking is observed at 0.10 strain in the INPG sample (Figure 6(d3)). At 0.16 strain, extensive ligament fracture has already occurred in ANPG, with shear deformation zones concentrating around these fracture sites (Figure 6(c4)). In contrast, INPG exhibits no ligament fracture under equivalent strain conditions (Figure 6(d4)). As deformation progresses, the continuing necking and eventual fracture of ligaments generate intense plastic strain accumulation in these critical areas, visually represented by the red-colored atoms in the strain mapping of Figure 6(c5,d5). The deformation analysis in Figure 6 reveals that plastic deformation in ANPG and INPG is not confined to eventual fracture surfaces alone. Significant plastic strain distribution occurs extensively in regions far from the primary fracture path, demonstrating that these materials undergo substantial plastic redistribution before final failure. This widespread plastic activity enables the structure to fail through a ductile fracture mechanism rather than experiencing abrupt brittle failure, highlighting the enhanced damage tolerance inherent to nanoscale porous metallic materials. It is noteworthy from Figure 6 that both ANPG and INPG exhibit a consistent ligament fracture pattern characterized primarily by axial rupture along the tensile direction, with the final fracture surfaces oriented approximately perpendicular to the applied tensile axis. This phenomenon can be attributed to the structural realignment mechanism during tensile deformation, where ligaments undergo progressive reorientation toward the loading direction. As the strain increases, many initially random or preferentially aligned ligament structures tend to align with the tensile axis, creating favorable conditions for crack propagation along the reoriented ligament pathways. The strain localization can serve as a predictor of where the ligaments will eventually rupture. The resulting fracture morphology demonstrates how the inherent anisotropy of nanoporous structures governs the damage evolution process, with the final fracture plane developing normal to the principal stress direction regardless of the initial ligament orientation.

**Figure 7:**
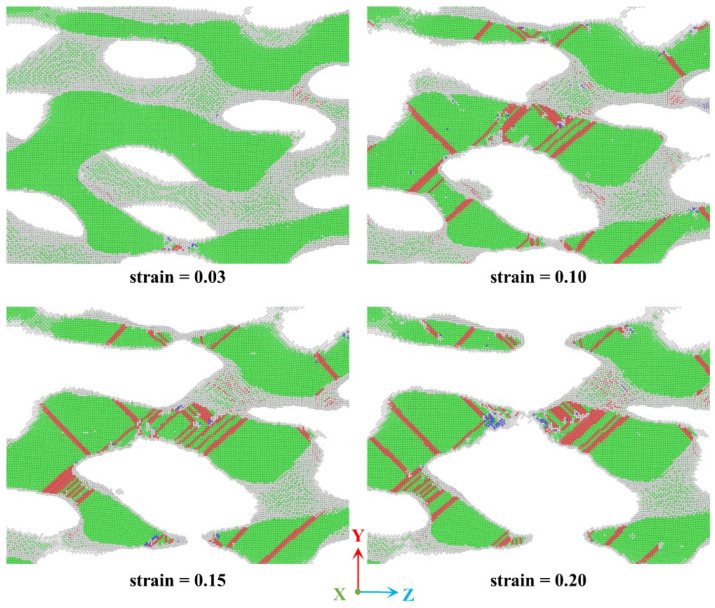
Local microstructural change in selected regions of ANPG with a relative density of 0.4 during tensile deformation. Atoms are color-coded based on their lattice types: green represents FCC-structured Au atoms, white corresponds to amorphous atoms (surface atoms), red indicates HCP-structured atoms, and blue denotes BCC-structured atoms.

For clearer observation of plastic events during tensile deformation, we perform a detailed analysis of a local region within the ANPG structure with a 0.4 relative density, as shown in the corresponding CNA results in Figure 7. The analysis reveals multiple plastic deformation mechanisms, including Shockley partial dislocation nucleation and propagation, the formation of stacking faults (comprising both intrinsic and extrinsic types), migrating twin boundaries, and the localized emergence of BCC and HCP structures. Extensive dislocation, cross-slip, and interactions lead to progressive ligament necking, ultimately resulting in fracture. Dislocation glide occurs preferentially along the {111} close-packed planes in the <110> direction, following the primary slip system inherent to face-centered cubic (FCC) crystal structures. The final fracture morphology indicates that ligaments predominantly fail along the tensile direction. In addition, the formation of Lomer-Cottrell locks is observed during plastic deformation. These sessile dislocation structures effectively impede the glide of mobile dislocations, thereby enhancing the resistance to external loads and elevating the overall stress level required for continued deformation. These observed plasticity mechanisms, including dislocation interactions, stacking fault formation, and twinning, are consistent with those reported in previous studies on conventional isotropic nanoporous metals [17,55,56].

**Figure 8:**
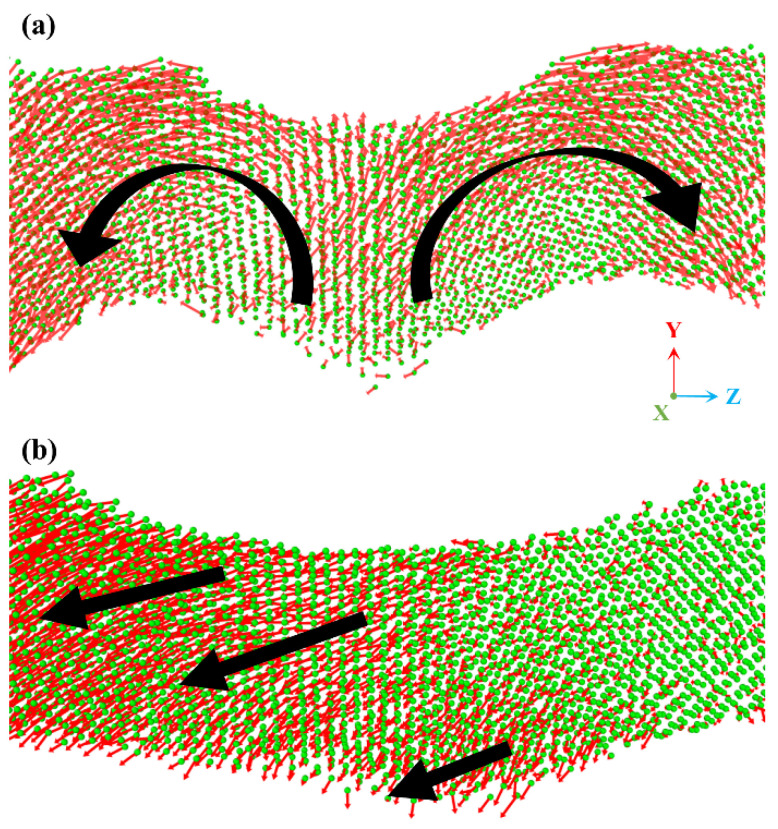
Local atomic displacement vector distribution within a representative ligament of (**a**) INPG and (**b**) ANPG at a tensile strain of 0.03 (elastic deformation stage). The red arrows indicate the displacement vector of each atom relative to its position in the undeformed state. The black arrows illustrate the motion trends of large atomic clusters.

Figure 8 provides direct atomic-scale visualization of the incipient deformation mechanisms of a single ligament in INPG and ANPG under elastic tensile strain (0.03). The red arrows indicate the displacement vector of each atom relative to its position in the undeformed state. The black arrows illustrate the collective motion of large atomic clusters. Based on the atomic displacement mapping presented in Figure 8, distinct deformation mechanisms can be identified between INPG and ANPG during the elastic regime. Specifically, remarkable differences in the direction and the extent of atomic motion are captured. For INPG, the local ligament deformation is dominated by bending and rotational modes, as evidenced by the black curved trajectories of atomic displacement vectors. In contrast, ANPG exhibits a coordinated deformation pattern combining axial stretching with minor bending components, where displacements preferentially align with the tensile direction despite minor deviation, indicating that axial deformation serves as the primary elastic mechanism. This mechanistic observation aligns consistently with our findings from the mechanical scaling analysis in Figure 4a, wherein the modulus-density relationship of ANPG deviates from classical bending-dominated models due to the significant contribution of axial stretching to overall stiffness. Therefore, it can be concluded that for ANPG, a greater proportion of ligaments undergo axial deformation rather than bending/rotational deformation, which facilitates more efficient load transfer and consequently enhances the global stiffness and strength. This observation and the inherent mechanisms align well with findings reported in previous studies [21,22].

**Figure 9:**
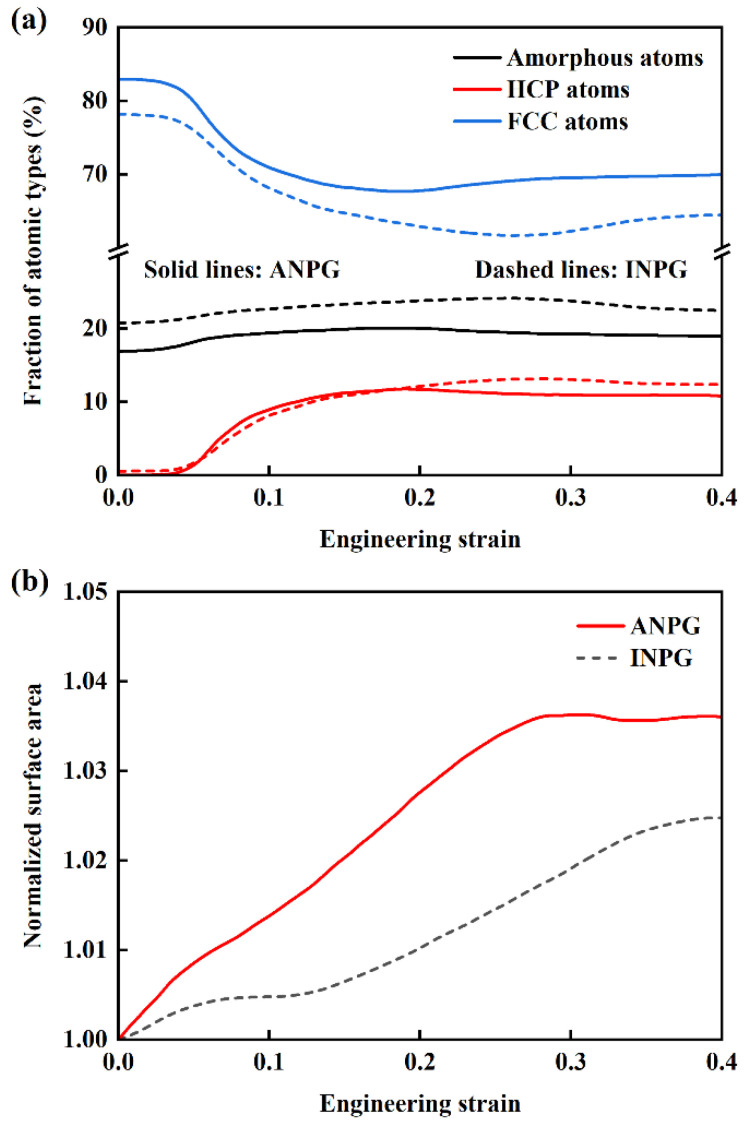
Variation in (**a**) fraction of atomic types and (**b**) normalized surface area with tensile strain for INPG and ANPG samples with a relative density of 0.40.

To quantitatively investigate the plastic deformation evolution in INPG and ANPG samples under tensile loading, we analyze the variations in different atomic types in both INPG and ANPG structures with a relative density of 0.40, as illustrated in Figure 9a. The black, blue, and red curves represent the percentages of amorphous, FCC, and HCP-structured atoms, respectively, with solid and dashed lines corresponding to ANPG and INPG. In the small-strain regime (below ~0.045), the fraction of HCP atoms remains negligible while the FCC percentage remains nearly constant, indicating pure elastic deformation without plastic activities. Notably, the initial FCC atom percentage in INPG is lower than in ANPG, which can be attributed to the longer and thicker ligaments aligned along the tensile direction in ANPG, resulting in a greater proportion of atoms located in the ligament interior rather than on the surfaces. Upon yielding (at ~0.045 strain), the HCP fraction increases pronouncedly, accompanied by a decrease in FCC atoms, signaling the onset of plastic deformation mechanisms including dislocation glide, stacking fault formation, and phase transformation. As strain increases, the fraction of HCP atoms progressively rises while the FCC atomic population correspondingly decreases. This increase in the fraction of HCP atoms implies the high toughness, which represents the enhancement of the energy absorption capacity during tensile deformation [16]. Beyond approximately 0.15 strain, the HCP atom population stabilizes, suggesting exhaustion of available dislocation sources and saturation of stacking fault generation. Subsequent deformation proceeds mainly through progressive ligament necking and fracture. While INPG and ANPG exhibit similar HCP fractions at strains below ~0.20, indicating comparable plastic deformation behavior, significant divergence emerges at higher strains. The higher HCP content in INPG beyond 0.20 strain reflects more extensive dislocation-mediated plasticity and stacking fault activity, whereas ANPG undergoes predominantly ligament necking and fracture, consistent with its earlier failure observed in Figure 3.

Figure 9a primarily reflects dislocation slip, stacking fault formation, and HCP phase transformation within the structure, yet provides limited insight into ligament necking behavior. To quantitatively evaluate this aspect, we calculate the structural surface area evolution. Figure 9b displays the normalized specific surface area as a function of tensile strain for INPG and ANPG with 0.40 relative density, where the specific surface area is normalized against the undeformed state. The increasing trend in specific surface area during tensile deformation arises from newly exposed surface atoms generated by ligament necking and fracture. The consistently higher specific surface area of ANPG compared to INPG confirms more intensive ligament necking and fracture events, correlating with its lower fracture strain.

**Figure 10:**
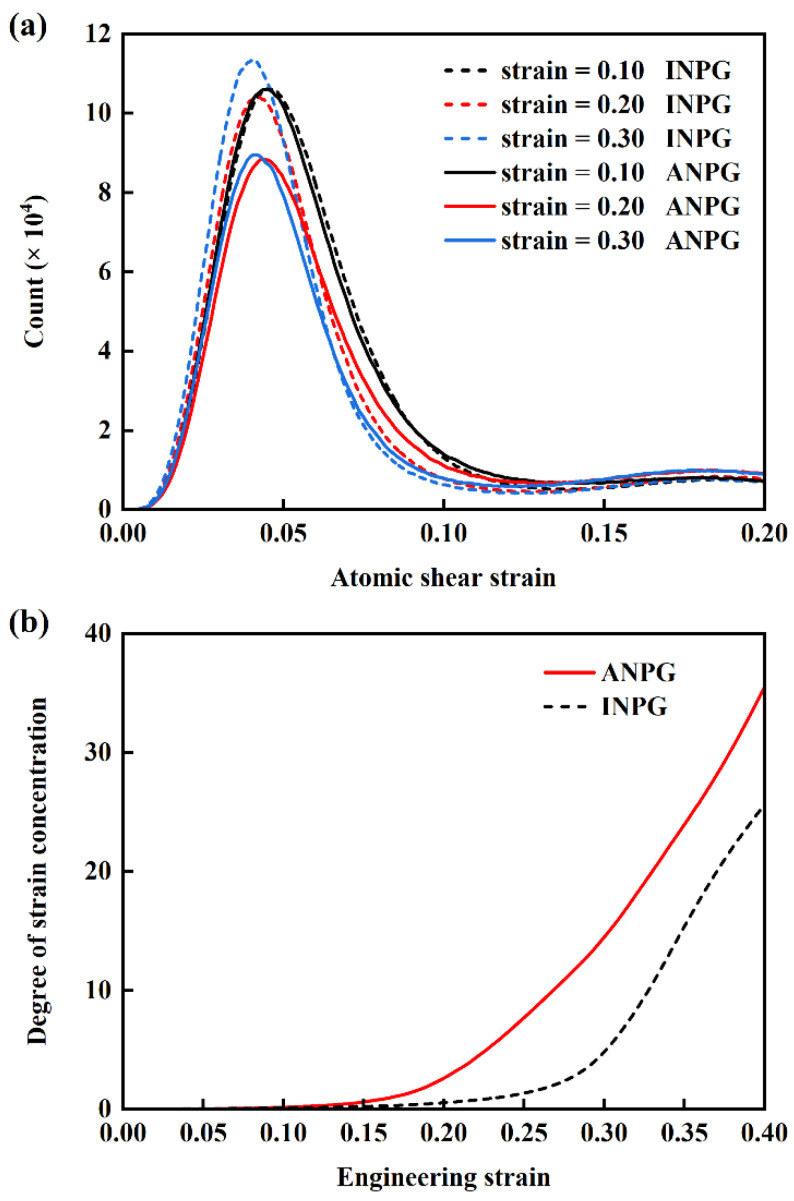
(**a**) Distribution of atomic shear strain at different global tensile strains for the ANPG (solid lines) and INPG samples (dashed lines) with 0.40 relative density. (**b**) Evolution of the degree of local deformation concentration with tensile engineering strain for the ANPG (solid lines) and INPG samples (dashed lines).

The statistical distribution of shear strain of individual atoms during plastic deformation is quantitatively characterized. Figure 10a presents the atomic shear strain distributions for both INPG and ANPG at a relative density of 0.40 under applied strains of 0.10, 0.15, and 0.20, with solid and dashed lines representing ANPG and INPG, respectively. At the relatively smaller global plastic strain of 0.10, despite the onset of plastic deformation, the shear strain distributions of ANPG and INPG remain nearly identical, as indicated by the overlapping black solid and dashed lines. However, as strain increases to 0.15 and 0.20, discernible differences emerge between the two structures. Particularly at 0.20 strain, ANPG exhibits a noticeably lower distribution peak and a broader strain spread compared to INPG. This indicates that in INPG, a larger proportion of atoms experience shear strains concentrated near the peak value, reflecting more homogeneous deformation, whereas ANPG develops a more heterogeneous strain distribution with increasing plastic deformation. To quantitatively investigate the strain localization extent, we calculate the deformation concentration degree using the formula:

(5) $$\psi =\sqrt{\frac{1}{N}\sum_{i}^{N}{\left(\eta_{\mathrm{Mises}}^{i}-\eta_{\mathrm{Mises}}^{\mathrm{ave}}\right)}^{2}}$$

where *N* is the total number of atoms of the INPG and ANPG sample, $\eta_{\mathrm{Mises}}^{i}$ is the von Mises shear strain of atom *i*, and $\eta_{\mathrm{Mises}}^{\mathrm{ave}}$ is the average von Mises shear strain of all atoms inside the sample. In fact, the quantified degree of strain localization is mathematically represented by the standard deviation of atomic shear strain. Figure 10b illustrates the variation in deformation concentration degree with engineering tensile strain for the INPG and ANPG samples with a relative density of 0.40. Evidently, before the 0.10 strain, the deformation concentration remains minimal and shows no significant difference between INPG and ANPG. Beyond 0.10 strain, the degree of deformation concentration increases remarkably with tensile strain, implying the generation of deformation localization. ANPG exhibits markedly higher deformation concentrations. This enhanced localization stems from its dominant deformation mechanism shifting toward ligament necking and fracture, instead of extensive dislocation-mediated plasticity. The resulting intensified strain localization promotes earlier failure and accounts for the lower fracture strain observed in ANPG.

Before conclusion, it is important to acknowledge that the remarkable mechanical enhancements and observed deformation behaviors reported here for ANPG are specific to the direction of ligament alignment (the out-of-plane direction). The mechanical responses under off-axis, transverse, or compressive loading could be substantially different and warrant future investigation. Furthermore, our model employs a single-crystal structure. It does not capture the potential influence of grain boundaries, which play a crucial role in the deformation and fracture of polycrystalline NPG [8,16]. Nevertheless, the present work provides fundamental insights into the anisotropic design principle, which remains highly relevant for numerous applications, such as linear actuators and membrane-based sensors [57], where the uniaxial mechanical responses are the primary mechanical consideration.

## 4. Conclusions

This study investigates the mechanical properties and deformation mechanisms of isotropic and anisotropic nanoporous gold (INPG and ANPG) through atomic-scale modeling and mechanical characterization. The distinctive feature of ANPG lies in its unique structural architecture with ligaments preferentially oriented along one specific direction, which gives rise to superior out-of-plane Young’s modulus and yield strength compared to traditional INPG, while maintaining comparable toughness to INPG at relatively low densities (below 0.4), demonstrating its potential as a high-performance structural material. Specifically, at relative densities of 0.30, 0.35, 0.40, 0.45, and 0.50, the out-of-plane Young’s modulus of ANPG increases by 84.8%, 89.2%, 73.7%, 53.6%, and 55.2%, respectively, compared to INPG. The corresponding yield strength enhancements are 43.8%, 39.1%, 31.0%, 31.4%, and 30.2%. The elastic deformation of ANPG is governed by a hybrid mechanism combining ligament bending and axial stretching, with stretching playing a relatively dominant role due to the unidirectional ligament structure. During plastic deformation, both ANPG and INPG exhibit similar dislocation-mediated plasticity in the early stages. However, in the later stages, ANPG shows an earlier transition in deformation mode: dislocation activity diminishes, giving way to more intensive ligament necking and fracture. This shift toward localized deformation explains the lower fracture strain observed in ANPG. The superior out-of-plane mechanical properties of ANPG make it particularly suitable for applications involving unidirectional loading scenarios, such as membrane actuators, force sensors, and protective coatings, where excellent mechanical performance along a specific direction is paramount. The tunable nature of its ligament architecture opens possibilities for designing graded structures with spatially varying anisotropy. Future work should explore multifunctional applications that leverage both its mechanical and transport properties, as well as develop controllable fabrication techniques to realize these designed architectures at macroscopic scales.

## Data Availability

The data that support the findings of this study are available from the corresponding author upon reasonable request.
